# Supplementary material for: Characterization of Neurons Expressing the Novel Analgesic Drug Target Somatostatin Receptor 4 in Mouse and Human Brains
Source: Int J Mol Sci. 2020 Oct 21;21(20):7788. doi: 10.3390/ijms21207788 (PMC7589422; doi:10.3390/ijms21207788)
Supplement: Supplementary file 1 [file ijms-21-07788-s001.pdf]

Supplementary Materials for ***Characterization of neurons expressing the novel analgesic drug target somatostatin receptor 4 in mouse and human brains***

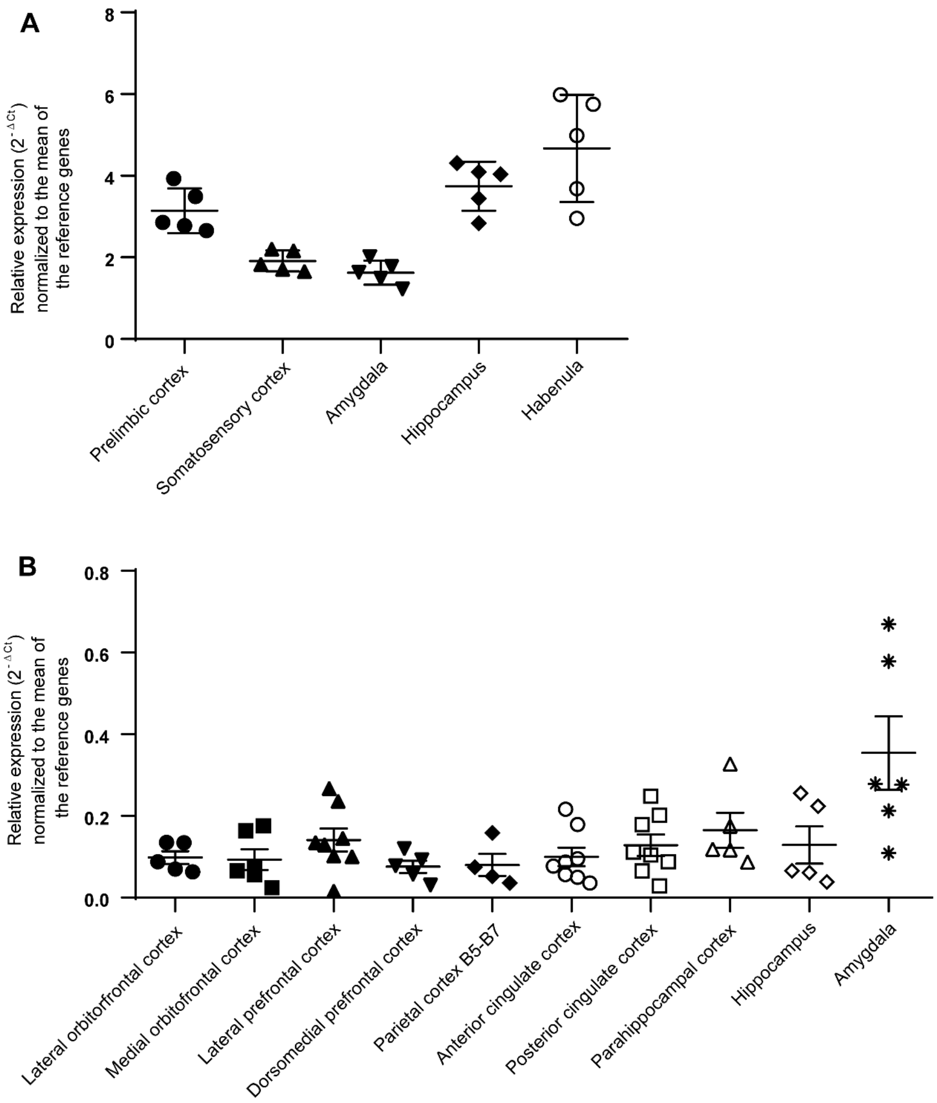

Figure S1. Relative mouse and human *Sstr4*/*SSTR4* mRNA were detected in various mouse brain micropunches (**A**) and human post-mortem cortical tissues (**B**) by RT-qPCR. Scattered dot plots represent SEM of $N = 5$ mouse and 4–8 human brain samples obtained from each brain areas. All qPCR experiments were performed in technical replicates. The geometric mean of the reference gene Ct values was determined and *Sstr4*/*SSTR4* mRNA expression relative to the reference genes (mouse *Gus* and *Hmbs*, and human *POLR2A*, *PES1* and *IPO8*) were calculated using the $2^{-\Delta Ct}$ formula to compare different brain regions.

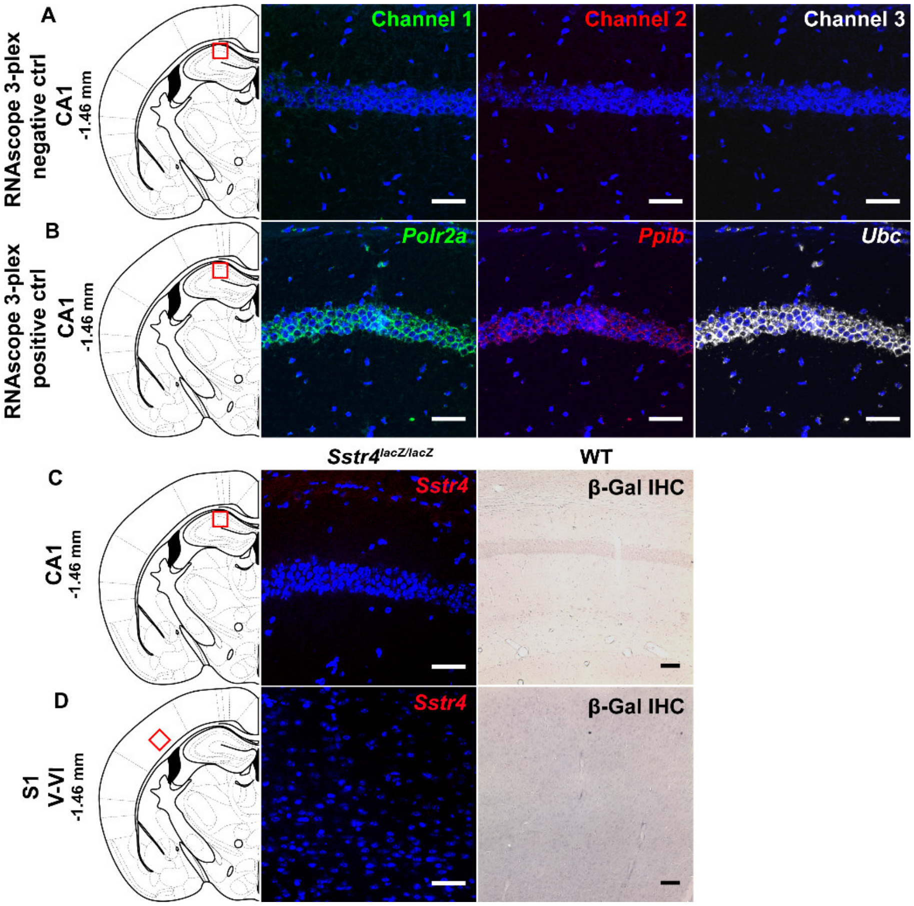

Figure S2. Representative control conditions for the mouse histological studies (by RNAscope counterstained with DAPI and β-Gal immunohistochemistry (IHC)). RNAscope 3-plex negative control specific to bacterial *dabP* gene are shown in the CA1 (**A**) from WT mice (*N* = 2). RNAscope 3-plex positive control probes specific to mouse *Polr2a*, *Ppib* and *Ubc* mRNA targets shown in green, red and white, respectively in the CA1 of the hippocampus (CA1, **B**) from WT animals (*N* = 2). No *Sstr4* signal is detectable either in the CA1 (**C**, left panel) or in the primary somatosensory cortex (S1, **D**, left panel) from *Sstr4*$^{lacZ/lacZ}$ mice by RNAscope (*N* = 2). Similarly, no *lacZ* signal is depicted either in the CA1 (**C**, right panel) or in the S1 (**D**, right panel) from WT mice by β-Gal IHC (*N* = 2). Scale bar: 50 µm.

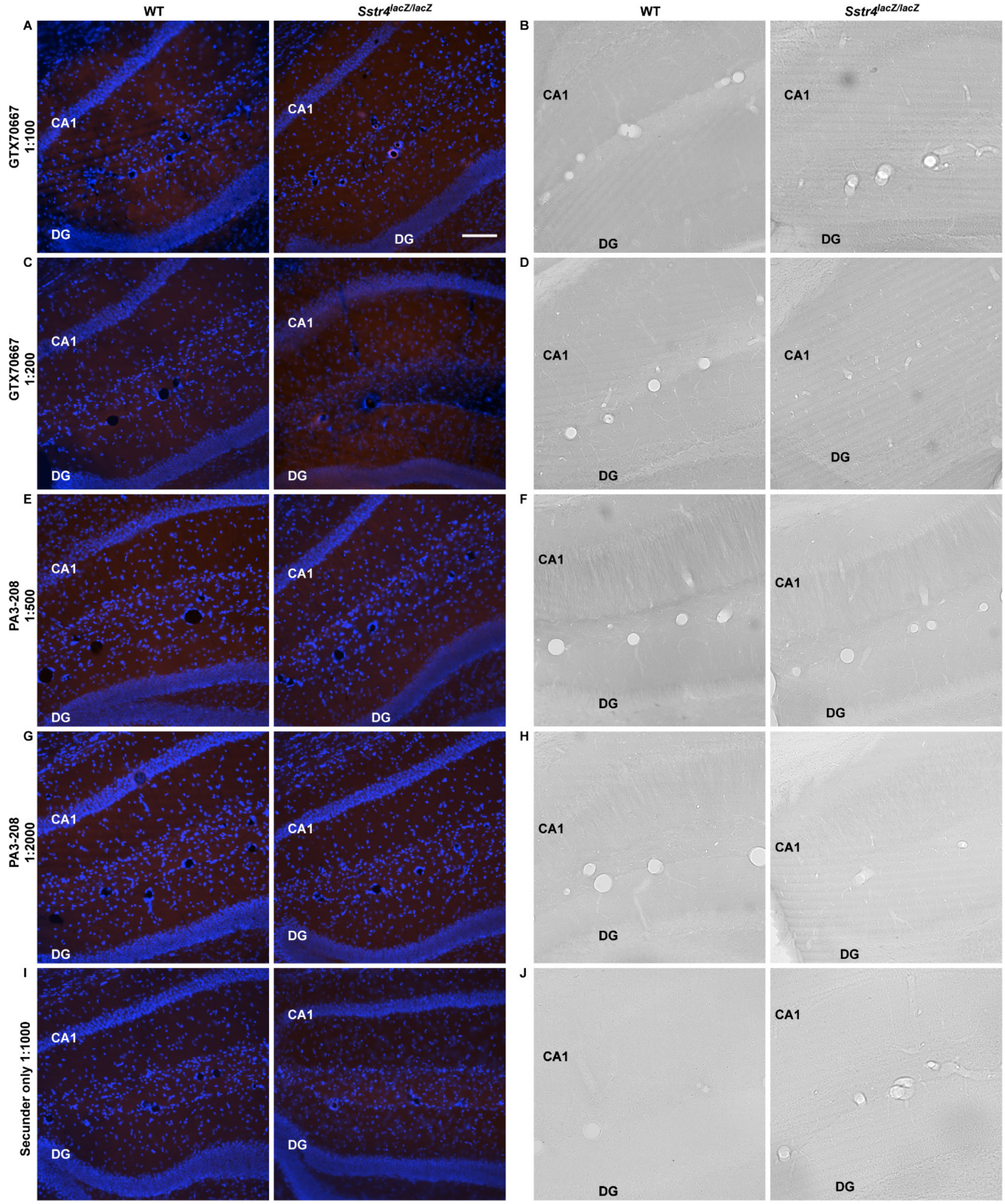

Figure S3. Representative images of IF and DAB-IHC staining on coronal sections (Bregma −1.4 mm) from C57BL6/J and *Sstr4*$^{lacZ/lacZ}$ *mice* (*n* = 2). **A-B**, sections were incubated with 1:100 diluted GTX70667 (GeneTex) anti-SST$_4$ antibody. **C-D**, sections were incubated with 1:200 diluted GTX70667 anti-SST$_4$ antibody. **E-F**, sections were incubated with 1:500 diluted PA3-208 (Thermo Fisher Scientific) anti-SST$_4$ antibody. **G-H**, sections were incubated with 1:2000 diluted PA3-208 anti-SST$_4$ antibody. **I**, sections were incubated with 1:1000 diluted goat anti-rabbit IgG secondary antibody

conjugated to Alexa Fluor 594 (A-11012, Thermo Fischer Scientific), no primary antibody control. **J**, sections were incubated with 1:200 diluted biotinylated goat anti-rabbit antibody, DAB signal was ABC method-amplified, no primary antibody control. Scale bar is 50 μm, same magnification was used on every image.

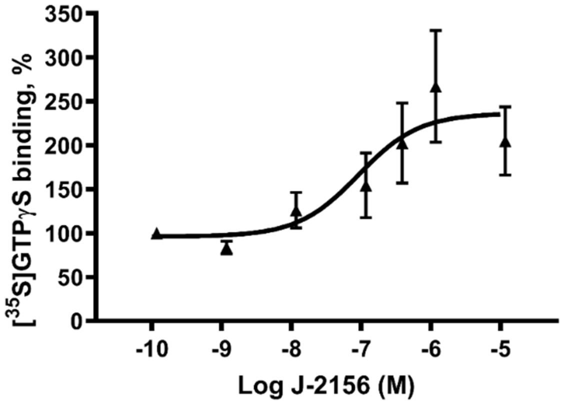

Figure S4. Effect of J-2156 on SST_4 receptor-linked G-protein activation. [^{35}S]GTPγS binding induced by J-2156 in CHO cells stably expressing the human SST_4 receptor. Each data point represents the mean±SEM of *n* = 3 experiments.

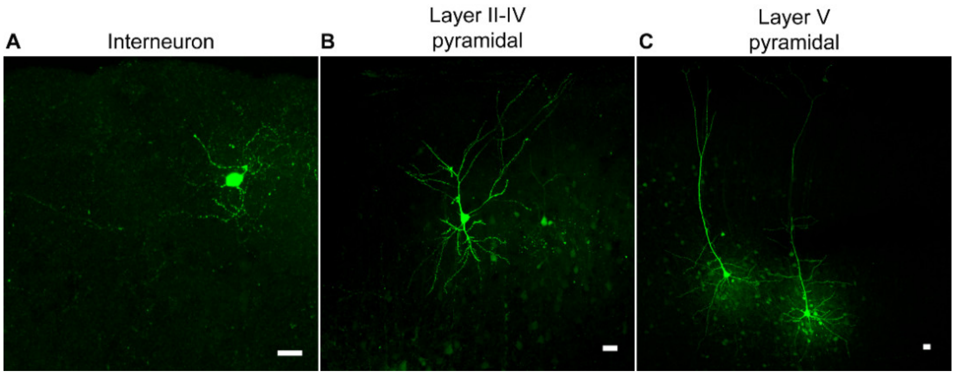

Figure S5. Biocytin filled and patch clamp recorded neurons from different layers of the somatosensory cortex. Representative confocal images of biocytin-filled interneuron (**A**), layer II–IV (**B**) and layer V pyramidal neurons (**C**) of the somatosensory cortex. Scale bar: 20 μm.

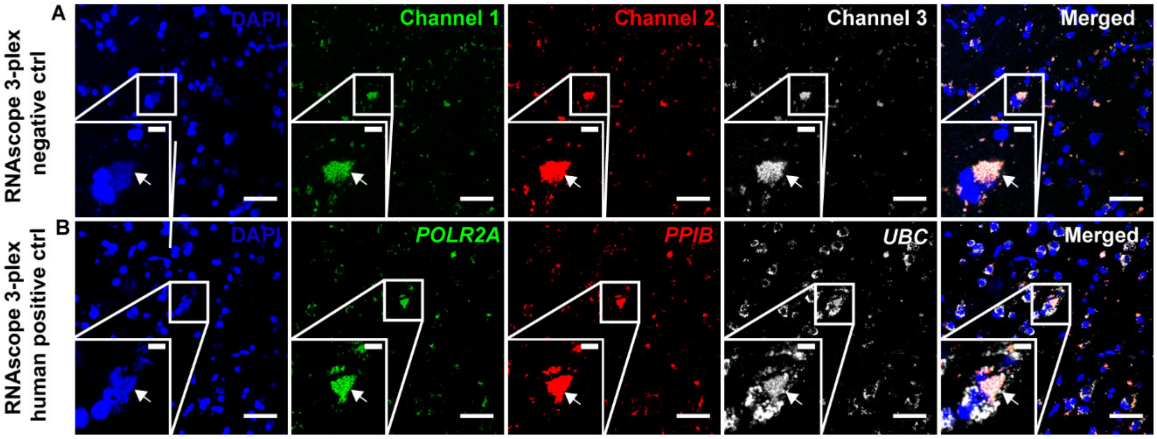

Figure S6. Representative control conditions for layer V pyramidal neurons of human neurosurgical cortical sample taken from the left middle temporal gyrus (by multiplex fluorescent RNAscope ISH). RNAscope 3-plex negative control probes specific to bacterial *dabP* gene (**A**) and RNAscope 3-plex positive control probes (**B**) specific to human *POLR2A* (green), *PPIB* (red) and *UBC* (white) mRNA targets are shown in layer V pyramidal neurons from Patient A (N = 1). Note that the tissue contains considerable amount of lipofuscin that shows some autofluorescence in all examined channels (arrows). Scale bar: 50 μm, inset scale bar: 10 μm.

Patient	Gender	Age	Post-mortem delay (h)	Died in	Others	Brain area
**1**	male	42	3.5	Cardiac insufficiency		Posterior cingulate cortex<br>Parahippocampal cortex<br>Hippocampus
**2**	female	79	4.5	Cardiac insufficiency		Parietal cortex B5-B7<br>Posterior cingulate cortex<br>Hippocampus
**3**	male	66	4.5	Cardio-pulmonal insufficiency	Leukemia	Parahippocampal cortex<br>Amygdala
**4**	female	48	5	Cardiac insufficiency		Lateral orbitofrontal cortex<br>Lateral prefrontal cortex<br>Anterior cingulate cortex suprag<br>Posterior cingulate cortex<br>Amygdala
**5**	male	52	4.5	Cardiac insufficiency		Lateral orbitofrontal cortex<br>Medialis orbitofrontal cortex<br>Lateral prefrontal cortex<br>Anterior cingulate cortex suprag<br>Posterior cingulate cortex<br>Dorsomedial prefrontal cortex
**6**	female	44	5	Cardiac insufficiency	Arterio-sclerosis	Parietal cortex B5-B7<br>Posterior cingulate cortex

Patient	Gender	Age	Post-mortem delay (h)	Died in	Others	Brain area
**7**	male	53	5	Pulmonal insufficiency		Parahippocampal cortex
**8**	male	63	3.5	Pulmonal insufficiency		Lateral orbitofrontal cortex<br>Dorsomedial prefrontal cortex<br>Anterior cingulate cortex supragenuculate<br>Posterior cingulate cortex<br>Parahippocampal cortex<br>Amygdala
**9**	male	61	5	Cardiac insufficiency	Diabetes	Medialis orbitofrontal cortex<br>Lateral prefrontal cortex<br>Dorsomedial prefrontal cortex<br>Posterior cingulate cortex<br>Anterior cingulate cortex suprag
**10**	male	27	8	Pulmonal insufficiency		Lateral orbitofrontal cortex<br>Lateral prefrontal cortex<br>Anterior cingulate cortex suprag<br>Hippocampus<br>Amygdala
**11**	female	26	6.5	Cardiac insufficiency		Medialis orbitofrontal cortex<br>Anterior cingulate cortex suprag<br>Hippocampus<br>Lateral prefrontal cortex<br>Parietal cortex<br>B5-B7

Patient	Gender	Age	Post-mortem delay (h)	Died in	Others	Brain area
**12**	male	50	5.5	Stroke		Hippocampus
**13**	male	55	2	Cardio-pulmonal insufficiency		Medialis orbitofrontal cortex<br>Lateral prefrontal cortex<br>Dorsomedial prefrontal cortex<br>Parietal cortex<br>B5-B7<br>Amygdala
**14**	male	68	10	Cardiac insufficiency	Arterio-sclerosis	Medialis orbitofrontal cortex<br>Anterior cingulate cortex suprag
**15**	female	72		Cardio-pulmonal insufficiency	Arterio-sclerosis	Medialis orbitofrontal cortex<br>Lateral orbitofrontal cortex<br>Lateral prefrontal cortex<br>Dorsomedial prefrontal cortex<br>Posterior cingulate cortex<br>Parahippocampal cortex<br>Amygdala

Table S1. Medical history of human patients (post-mortem samples)

Gene amplified	Nucleotide sequence of primer	Primer type	Product length in bp	NCBI RefSeq
**SYBR**				
**Mm *Gus***	CCGACCTCTCGAACAACCG	sense	169	NM_001357027.1
	GCTTCCCGTTCATACCACACC	antisense		
**Mm *Hmbs***	ATGAGGGTGATTCGAGTGGG	sense	134	NM_013551.2
	TTGTCTCCCGTGGTGGACATA	antisense		
**Mm *Sstr4***	GCCCTGGTCATCTTCGTGAT	sense	95	NM_009219.3
	ATGAAGAGCTCATCGGCGAC	antisense		
**PROBE**				
**Hs *PES1***	AGCGCCTGGCCATTATGA	sense	75	NM_001282327.1
	TTCGCCTCTTGCCAAACA	antisense		
	/56-FAM/ AGCGGGAGA/ ZEN/ AGTACCTGTACCAGA/3IABkFQ/	probe		
**Hs *IPO8***	ACCTTTGAAAACAAAGGAGTCC	sense	96	NM_001190995.1
	GCGATGACATTTGGTCAGCT	antisense		
	/5HEX/TTTCCTTCA/ZEN/GTTGTT GCTGGGCAC/3IABkFQ/	probe		
**Hs *POLR2A***	CCCCCAAAGGCTCAACCTAC	sense	83	NM_000937.5
	GATAGCCGGGCTTGTGAGAC	antisense		
	/5Cy5/TGTTTCCTGCACATGTTTG GCAGC/3IAbRQSp/	probe		
**Hs *SSTR4***	TGGAAGGTGCTGGAGGT	sense	126	NM_001052.2
	GTTCTGGTTGCAGGGCTT	antisense		
	/5Cy5/TGGACTACTATGCCACTGC TCTCAAGA/3IAbRQSp/	probe		

Table S2. Primers used to amplify target loci for RT-qPCR

Target	Catalog number	Fluorophores	Fluorophore dilution
Mm *Sstr4*	416641-C2	TSA Plus Cy3	1:750
Hs *SSTR4*	312851-C2	TSA Plus Cy3	1:750
Mm *Vglut1* (Slc17a7)	416631	TSA Plus Fluorescein	1:3000
Hs *VGLUT1* (*SLC17A7*)	415611	TSA Plus Fluorescein	1 1500
Mm *Chat*	408731	TSA Plus Fluorescein	1:1500
Mm *Gad1*	400951-C3	TSA Plus Cy5	1:3000
Mm *Rbfox3* (NeuN)	313311-C3	TSA Plus Cy5	1:750
Mm-3-plex Positive Control Probe	320881	TSA Plus Fluorescein, Cy3, Cy5	1:750
Hs-3-plex Positive Control Probe	320861	TSA Plus Fluorescein, Cy3, Cy5	1:750
3-plex Negative Control Probe	320871	TSA Plus Fluorescein, Cy3, Cy5	1:750

Mm-Mus musculus, Hs- Homo sapiens

Table S3. RNAscope probes

© 2020 by the authors. Licensee MDPI, Basel, Switzerland. This article is an open access article distributed under the terms and conditions of the Creative Commons Attribution (CC BY) license (http://creativecommons.org/licenses/by/4.0/).
